# Clinicopathological and molecular study of 10 salivary gland clear cell carcinomas, with emphasis on rare cases with high grade transformation and occurring in uncommon sites

**DOI:** 10.1186/s13000-022-01200-z

**Published:** 2022-01-30

**Authors:** Lanlan Xuan, Suxia Wang, Jianguo Wei, Jianwei Yuan, Honggang Liu

**Affiliations:** 1 Department of Pathology, Anqing Hospital, Anhui Medical University, Anqing Municipal Hospital, Anqing, 246003 China; 2 Department of Pathology, Beijing Tongren Hospital, Capital Medical University, Beijing Key Laboratory of Head and Neck Molecular Diagnostic Pathology, Beijing, 100730 China; 3 Department of Pathology, Yantai Yuhuangding Hospital of Qingdao University, Yantai, 264000 Shandong China; 4 Department of Pathology, Shaoxing People’s Hospital, Shaoxing, 312000 China; 5 Department of Oncology Surgery, Anqing Hospital, Anhui Medical University, Anqing Municipal Hospital, Anqing, 246003 China

**Keywords:** Clear cell carcinoma, Hyalinizing, High-grade transformation, *EWSR1*, *ATF1*, *MAML2*

## Abstract

**Background:**

As a rare salivary gland malignancy, clear cell carcinoma (CCC) is easily misdiagnosed. This study identified the features that allow better recognition of the clinicopathological and molecular characteristics and the prognosis of CCC, focusing on high-grade transformation (HGT) in this tumor and cases arising in uncommon sites.

**Methods:**

Clinicopathological and follow-up data for 10 CCC samples were retrieved. Immunohistochemical (IHC) staining was performed, and fluorescence in situ hybridization (FISH) was used to detect *EWSR1* gene rearrangements, *EWSR1–ATF1* gene fusions, and *MAML2* gene rearrangements.

**Results:**

Histologically, typical CCCs comprised bland polygonal or round cells with clear cytoplasm. In contrast with typical CCCs, HGT tumor cells exhibited nuclear pleomorphism, high nuclear-to-cytoplasmic ratios, high mitotic activity, and necrosis. Rare morphologic features such as pseudopapillae, gland-like spaces, and entrapped ducts were also observed. Occasionally, tumors involving the oral cavity might arise from the overlying epithelium of the mucosal surface. Immunohistochemically, all the cases expressed p63, p40, and CK5/6, while myoepithelial-related markers were uniformly negative in all cases. HGT exhibited a wild type p53 expression pattern. FISH demonstrated *EWSR1* rearrangement (10/10) and *EWSR1–ATF1* fusion (4/5); however, *MAML2* remained intact (0/3).

**Conclusions:**

CCCs with HGT or occurring in uncommon sites are extremely rare. Combining morphology based IHC and molecular detection provided reliable evidence that the HGT component represented a transformation of CCC rather than the coexistence of another tumor and helped differentiating CCCs in uncommon sites from their mimics, avoiding potential misdiagnosis and inappropriate therapy. The overall prognosis for CCCs is good, except for the HGT cases, which needed continued treatment.

## Background

Clear cell carcinoma (CCC) is a low-grade salivary gland tumor (SGT), first described by Milchgrub et al. in 1994 from a series of 11 cases. CCC has the characteristic morphology of bland monomorphic tumor cells with clear cytoplasm, arranged in nests, islands, and sheets in a hyalinized stoma, which is why it was originally termed “hyalinizing clear cell carcinoma” (HCCC) [1]. CCC is typically present in the minor salivary glands of female patients aged 50–80 years; it is indolent and rarely involves lymph nodes or develops distant metastases [2]. Over the past several years, the pathology of SGTs has become widely studied due to the interesting molecular alterations involved, and molecular genetic techniques are being increasingly utilized by pathologists in the diagnoses of SGTs. Studies have revealed that a majority of CCCs harbor a translocation t (12;22)(q13;q12), resulting in the fusion of the *EWSR1* gene (22q12) with the *ATF1* gene (12q13), which in turn generates the chimeric gene *EWSR1–ATF1* [3–5] that has become a hallmark molecular event for this tumor.

High grade transformation (HGT) refers to the transformation of tumor cells with conventional morphology into poorly differentiated or undifferentiated tumor cells with high grade morphology. In other words, HGT is the transformation of conventional morphology into high-grade morphology, making the tumor becomes more aggressive. CCCs with HGT are extremely rare and seldom reported in the literature [6, 7], and few, if any, features associated with HGT are currently recognized in CCCs. Therefore, it is challenging to identify CCCs with HGT from other high-grade SGTs based on morphology alone. In addition, it is difficult to definitively diagnose CCCs found in uncommon sites due to the lack of distinguishing characteristics; misdiagnosing CCCs in uncommon sites as other malignancies with clear cell morphologic changes is common.

Here, we present a series of 10 new cases of CCC, including one HGT case and three cases occurring in uncommon sites (nasal cavity, tonsil, and maxillary sinus), followed by a literature review. The focus is on clinicopathological and molecular detection and their outcomes, paving the way for CCC recognition, diagnosis, and differential diagnosis in routine practice, with the aim of enhancing the understanding and treatment of this rare malignancy.

## Materials and methods

### Case selection

We searched for a total of 1669 cases of SGTs using the files of the SGT Registry at the Department of Pathology in Beijing Tongren Hospital, of which 10 cases pertained to CCC, accounting for 0.6%. Among the 10 cases of CCC, six were in-house cases and four were consultation cases. The four referred cases had been submitted with preliminary diagnoses to be confirmed; of these, three cases were originally considered to be mucoepidermoid carcinoma (MEC), and one case was originally considered a myoepithelial carcinoma (MC). Available clinicopathological data of these patients were retrieved, and cases were reviewed by two pathologists (X.L.L. and L.H.G.) specializing in salivary gland neoplasms. Follow-up data were obtained via telephonic medium. This study was approved by the Institute Research Ethics Committee of Beijing Tongren Hospital (Approval No. TRECKY2021–108), and written informed consent was obtained from 9 of 10 patients, whereas consent from one patient was not obtained because of loss to follow-up.

### Immunohistochemistry

All tissue samples had been fixed with 3.7% neutral formaldehyde and then embedded in paraffin. Samples were stained with hematoxylin and eosin and automated immunohistochemistry (IHC) staining was performed on 4 μm-thick formalin-fixed paraffin-embedded (FFPE) sections using the Benchmark XT platform (Ventana Medical Systems Inc., Tucson, AZ) according to the manufacturer’s instructions. Primary antibodies against the following proteins were purchased from Fuzhou Maixin Biotechnology Development Company (Fuzhou, China): cytokeratin 5/6 (CK5/6; clone D5/16B4), p63 (clone 4A4), p40 (clone Np63), smooth muscle actin (SMA; clone 1A4), calponin (clone CALP), S-100 protein (clone 4C4.9), SOX10 (clone EP268), GFAP (clone GA-5), CD10 (clone 56C6), CD117 (clone 2E4), p16 (clone 6H12), p53 (clone MX008), and Ki-67 (clone MIB-1). CK5/6, SMA, calponin, and GFAP positivity were defined as tumor cells exhibiting cytoplasmic staining, whereas p63, p40, SOX10, p53, and Ki-67 positivity were assessed by nuclear staining, S-100 protein and p16 positivity by nuclear and cytoplasmic staining, and CD117 positivity by cytoplasmic and/or membrane staining. Less than 80% of the nuclei exhibited p53 immunoreactivity with variable staining intensities, and this was considered to be the wild type expression pattern [8]. All primary antibodies were ready-to-use and did not require additional dilutions. Immunohistochemical staining was performed using the MaxVision method. The FFPE sections were subjected to EDTA antigen retrieval followed by the addition of a primary antibody, incubation at 37 °C for 45 min and rinsing with PBS. Then, the corresponding secondary antibody (MaxVision) was added to the sections, which were then incubated at 37 °C for 15 min and rinsed with PBS. Next, DAB development was carried out for 5 min. For all IHC stains, positive and negative controls were used. The staining results were assessed by two independent pathologists.

### Fish

A commercial *EWSR1* break-apart probe, an *MAML2* break-apart probe, and an *EWSR1–ATF1* fusion probe (all from LBP Medicine Science and Technology Co., Ltd., Guangzhou, China) were used on FFPE sections. The experimental procedures were performed following manufacturer’s instructions. Two hundred non-overlapping tumor cell nuclei were observed by fluorescence microscopy (BX51; Olympus, Tokyo, Japan) for quantitative analysis. *EWSR1* and *MAML2* break-apart probes were considered to respond positively if > 15% of tumor cell nuclei exhibited red-green split signals (> two signal diameters apart). The presence of an isolated single red signal with a normal red-green signal (unsplit pair) was also counted as a gene rearrangement [9, 10]. *EWSR1–ATF1* fusion was considered positive if > 15% of tumor cell nuclei exhibited at least one yellow (red-green fusion) signal. Signals were assessed independently by two technicians.

## Results

### Clinical finding

Clinical data from the cohort are shown in Table 1. All cases were primary and included six female and four male individuals, ranging in age from 29 to 70 years (mean = 46 years). Four CCCs occurred on the palate and one each from the left tonsil, the left maxillary sinus, root of the tongue, base of the tongue, the nasal cavity, and the submandibular gland. The main clinical symptoms included the development of a painless firm mass, epistaxis, paresthesia, ulceration, and dysphagia.

**Table 1:** Clinicopathological data for 10 CCCs

Case	Sex/Age (y)	Site	Maximum size (cm)	Clinical presentation	Follow-up (months)
1*	F/68	Right submandi-bular gland	4.0	Painless firm mass, recently enlarged with pain	Alive with tumor (7)
2	F/70	Nasal cavity	2.0	Nasal obstruction and epistaxis	Alive NED (80)
3	M/48	Left tonsil	3.5	Pharyngeal paresthesia	Alive NED (17)
4	M/32	Left maxillary sinus	2.5	Epistaxis	Alive NED (88)
5	F/62	Root of the tongue	3.0	Dysphagia	Alive NED (23)
6	F/31	Base of tongue	3.2	Dysphagia	Alive NED (15)
7	M/29	Right hard palate	2.3	Painless firm mass	Alive NED (8)
8	M/29	Right hard palate	1.8	Ulceration	Alive NED (11)
9	F/49	Right hard palate	2.2	Ulceration	NA
10	F/37	Left hard palate	1.2	Ulceration	Alive NED (54)

*High-grade transformation (HGT) case

*F* female, *M* male, *NED* no evidence of disease, *NA* not available

Among the cases analyzed, cases 3 (left tonsil), 5 (base of tongue), and 10 (left hard palate) documented neck lymph node dissection, and cases 3 and 10 recorded lymph node metastasis (2/15 and 1/16, respectively), confirmed by pathological examinations.

**Fig. 1:**
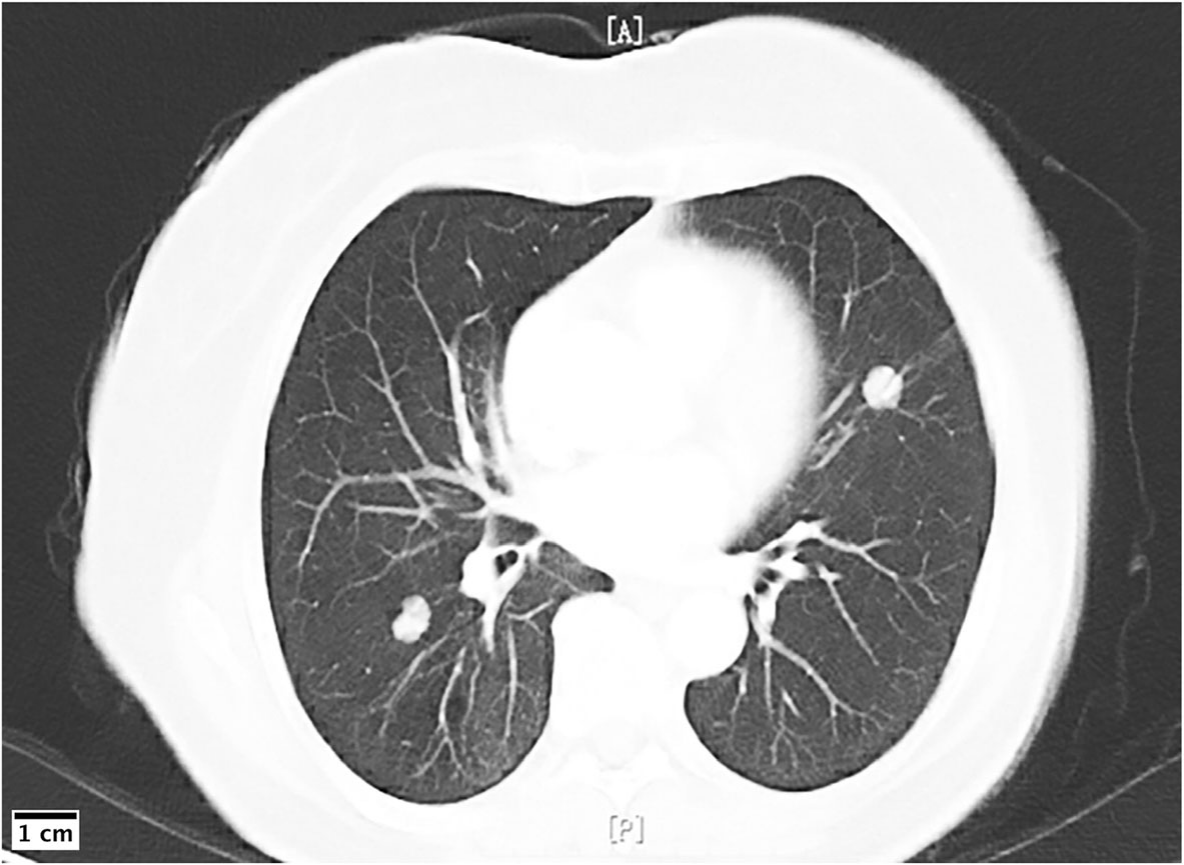
A computerized tomography chest scan showing multiple bilateral pulmonary nodules (Case 1)

Follow-up information was available for 9 patients, ranging from 7 to 80 months (mean, 33.7 months) after discharge. In Case 1, the patient was a 62-year-old woman with a 20-year history of a painless right submandibular gland mass that enlarged rapidly, causing pain during the last four months. A computed tomography chest scan at presentation showed multiple bilateral pulmonary nodules (Fig. 1). The patient underwent wide resection of the right submandibular mass, followed by chemotherapy, and was alive with the tumor at the time of writing this paper. Patients of cases 4, 8, and 10 received radiotherapy after surgery and were free of disease at follow-ups of 88, 11, and 54 months, respectively. Five patients did not undergo postoperative radiotherapy or chemotherapy, and none had recurrence or distant metastases at the last follow-up. One patient was lost to follow-up.

### Pathological features

**Fig. 2:**
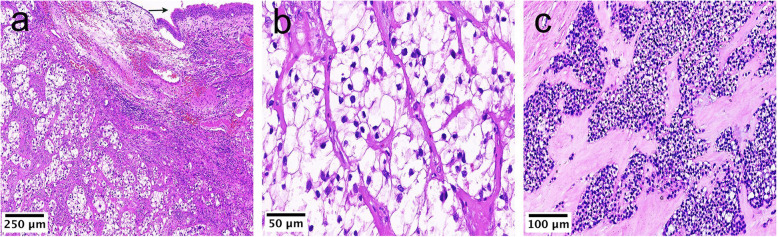
The typical histological morphology for CCC. (**a**) Ciliated columnar epithelium of the respiratory tract is observed at the upper right margin (arrow). The CCC was located beneath the mucosa (Case 2, HE × 100). (**b**) Tumors were typically composed of oval, round, and polygonal cells with clear cytoplasm, arranged in nests or sheets, surrounded by fibrocellular stroma (Case 2, HE× 400). (**c**) In the conventional low-grade region of the HGT case, nuclei were uniform and small, round or oval, and had irregular, indented contours with finely dispersed or dark condensed chromatin (Case 1, HE× 200)

Tumor sizes ranged from 1.0 cm to 4.0 cm (median, 2.4 cm; mean, 2.6 cm), as measured along the longest axis. Tumors were firm with gray-white or tan-white cut surfaces and were relatively circumscribed but lacked encapsulation. Histologically, conventional CCCs were composed of oval, round, and polygonal cells with clear cytoplasm (Case 2; Fig. 2a, b), although some tumor cells had pale eosinophilic cytoplasm (the conventional region of Case 1; Fig. 2c). The nuclei were uniform and small, round, or oval and had irregular, indented contours with dark, condensed chromatin, and nucleoli were typically not prominent (Case 2; Fig. 2b). Tumor cells were typically arranged in nests, cords, or sheets.

**Fig. 3:**
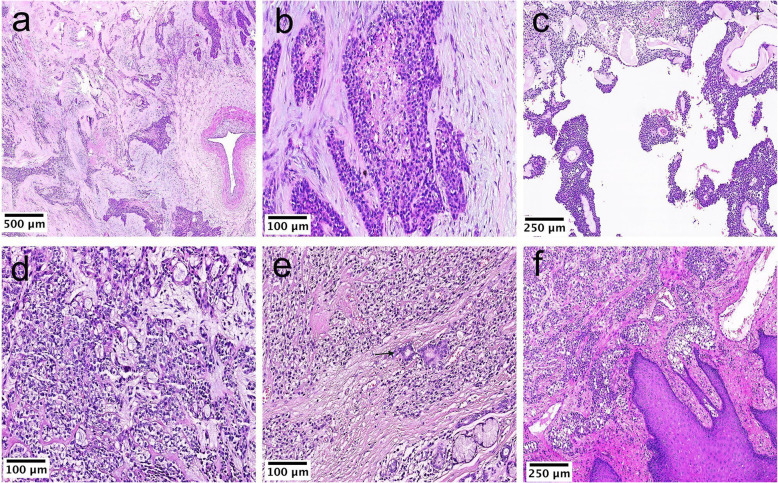
Uncommon histological morphology in CCC. (**a**) The morphological contrast between the conventional low-grade region on the left and the HGT region on the right and the middle (Case 1, HE× 50). (**b**) The HGT area showing nuclear pleomorphisms, high nuclear-to-cytoplasmic ratios, high mitotic activity, and necrosis (Case 1, HE× 200). (**c**) Cystic changes were observed within the tumor nest, forming pseudopapillae (Case 1, HE× 100). (**d**) Gland-like spaces were focally identified (Case 5, HE× 200). (**e**) Entrapped non-neoplastic ducts (arrow) (Case 6, HE× 200). (**f**) Tumor cells were connected to the surface overlying epithelium (Case 7, HE× 100)

However, we also observed some rare morphologies. In contrast to classic CCCs, HGT tumor cells exhibited nuclear pleomorphism, high nuclear-to-cytoplasmic ratios, high mitotic activity, and necrosis (Case 1; Fig. 3a, b), suggesting a poorly differentiated morphology. Cystic changes with pseudopapillary formation (Case1; Fig. 3c) and gland-like structures (Case 5; Fig. 3d) were observed within the tumor nests. Glands were also seen, although such structures were entrapped in non-neoplastic ducts (Case 6; Fig. 3e). In addition, tumors in oral sites were potentially connected to the mucosal surface epithelium and exhibited squamous epithelial hyperplasia (Case 7; Fig. 3f).

**Fig. 4:**
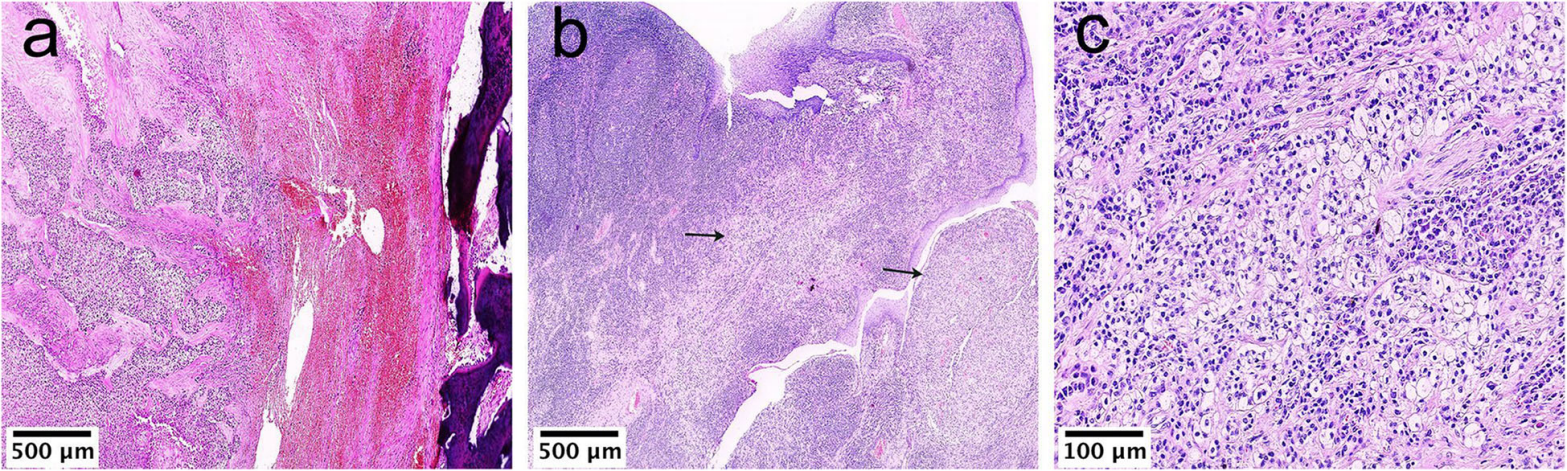
Uncommon stromal alterations in CCC. (**a**) Stromal hemorrhage (Case 4, HE× 50). (**b**, **c**) Lymphoid infiltration around the tumor nests (the arrows indicate tumor cells) (Case 3, HE× 50 and HE× 200)

The tumor cells were separated by stroma that appeared hyaline (Case 1; Fig. 2c), fibrocellular (Case 2; Fig. 2a), and myxoid (Case 5; Fig. 3d). Occasionally, hemorrhages (Case 4; Fig. 4a) and lymphoid infiltration (Case 3; Fig. 4b, c) were also observed around the tumor nests.

### Immunohistochemical results

**Fig. 5:**
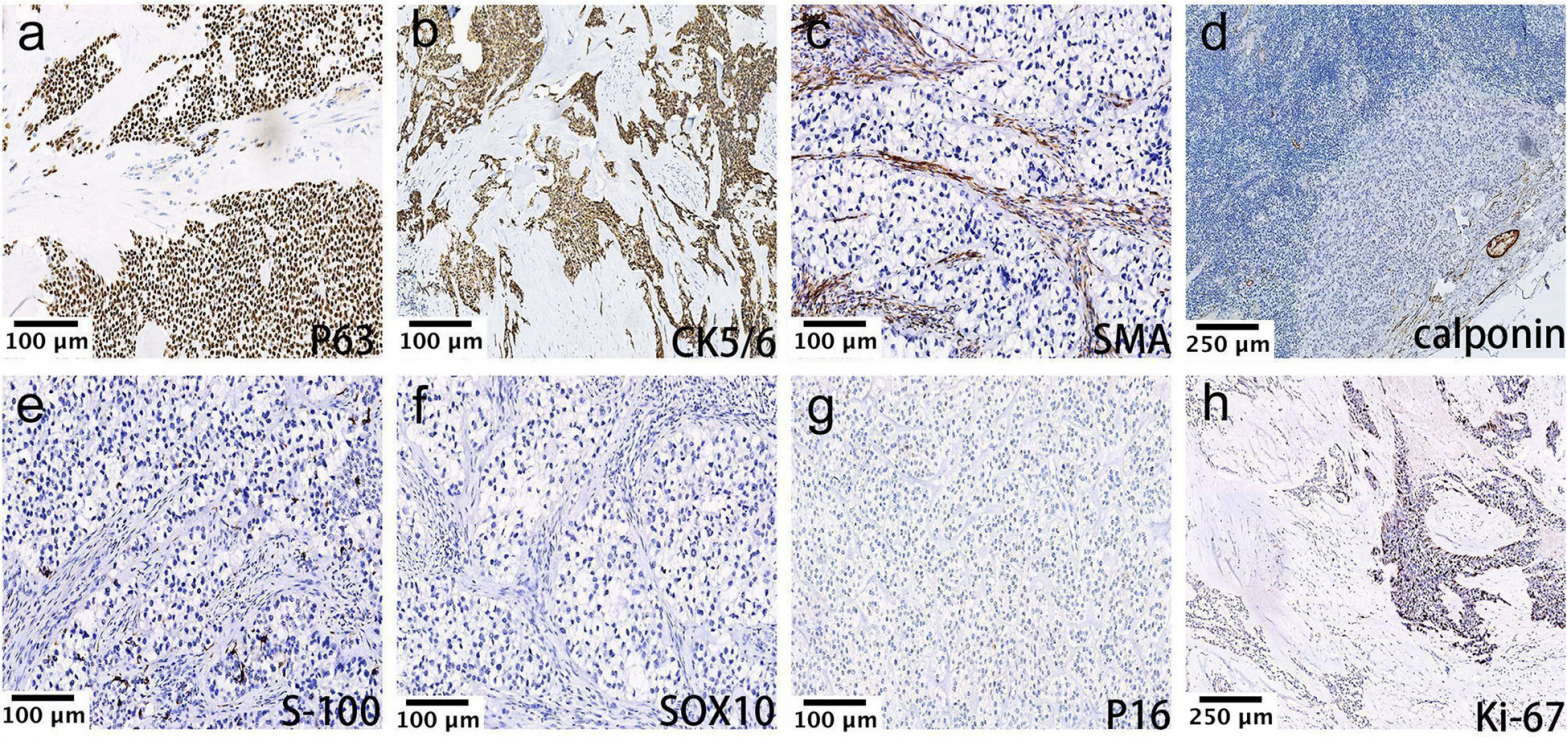
IHC for the CCC. The tumor cells were positive for (**a**) p63 (Case 1, IHC × 200) and (**b**) CK5/6 (Case 1, IHC × 200), and negative for (**c**) SMA (Case 2, IHC × 200), (**d**) calponin (Case 3, IHC × 100), (**e**) S-100 protein (Case 2, IHC × 200), and (**f**) SOX10 (Case 2, IHC × 200). (**g**) p16 expression was negative (Case 3, IHC × 200). (**h**) Different Ki-67 immunoreactivity was noted between the conventional region in the lower left corner (median, 1.9%) and the HGT region in the upper right corner (median, 42.5%) (Case 1, IHC × 100)

The IHC detection results are shown in Table 2. All the cases consistently expressed p63 (Case 1; Fig. 5a), p40, and CK5/6 (Case 1; Fig. 5b), while myoepithelial markers were uniformly negative, including SMA (Case 2; Fig. 5c), calponin (Case 3; Fig. 5d), S-100 protein (Case 2; Fig. 5e), GFAP, and CD10. SOX10 (Case 2; Fig. 5f) was also consistently negative in all cases. In Case 3, the left tonsil case, p16 was negative (Case 3, Fig. 5g). In Case 1, p53 showed a wild-type expression pattern in the HGT region, like the conventional region (not shown). CCCs from the nasal cavity, left tonsil, and left maxillary sinus (Cases 2, 3, and 4, respectively) were also detected with a large immunostaining panel (not shown) to confirm the diagnosis.

**Table 2:** IHC detection results in 10 cases of CCC

NO	Site	p63	p40	CK5/6	SMA	Calponin	S-100	GFAP	CD10	SOX10	p16	p53
1	Right submandibular gland	+	+	+	–	–	–	–	–	–	ND	W-T
2	Nasal cavity	+	+	+	–	–	–	–	–	–	ND	ND
3	Left tonsil	+	+	+	–	–	–	–	–	–	–	ND
4	Left maxillary sinus	+	+	+	–	–	–	–	–	–	ND	ND
5	Root of the tongue	+	+	+	–	–	–	–	–	–	ND	ND
6	Base of tongue	+	+	+	–	–	–	–	–	–	ND	ND
7	Right hard palate	+	+	+	–	–	–	–	–	–	ND	ND
8	Right hard palate	+	+	+	–	–	–	–	–	–	ND	ND
9	Right hard palate	+	+	+	–	–	–	–	–	–	ND	ND
10	Left hard palate	+	+	+	–	–	–	–		–	ND	ND

*IHC* Immunohistochemistry, *ND* not done, *W-T* wild-type expression

Ki-67 proliferation index was calculated for each field. Ten high-power fields (HPF) were arbitrarily selected and the median value was calculated. In the CCC case with HGT (Case 1), where staining was performed, there was a striking distinction between the Ki-67 index for the low-grade versus the high-grade components, with a low index (median, 1.9%) in the former and a high index (median, 42.5%) (Fig. 5h) in the latter.

### FISH detection results

**Fig. 6:**
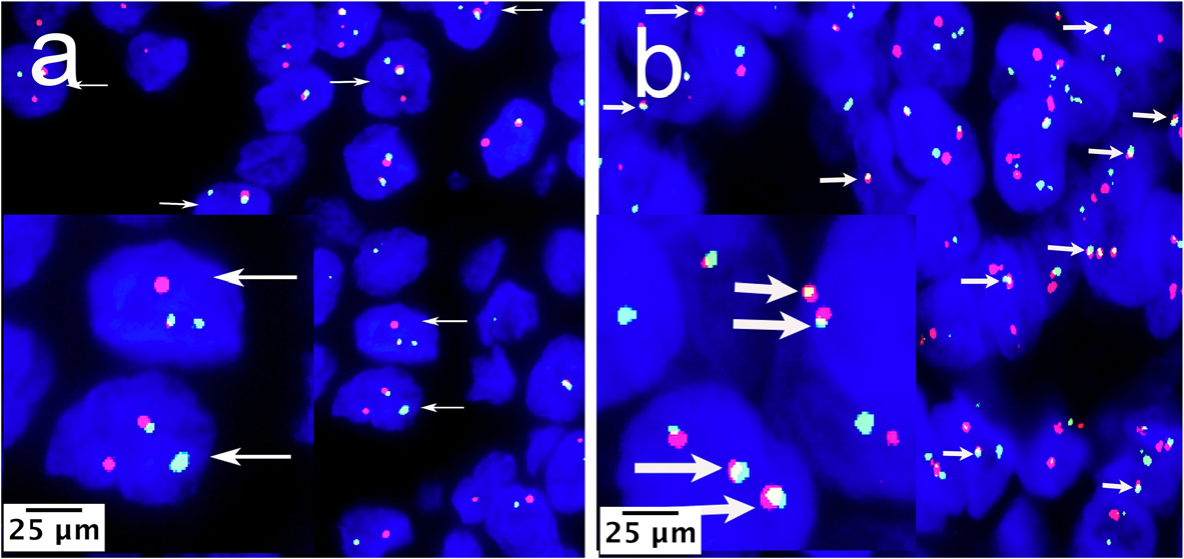
FISH for CCC. (**a**) FISH with *EWSR1* break-apart probe. The break-apart probe shows one separate red and green signal per cell (arrows) was considered positive for *EWSR1* rearrangement; the fused red-green (yellow) signal was considered normal (Case 1, FISH× 1000). (**b**) FISH with *EWSR1-ATF1* fusion probe. Showing at least one red-green (yellow) fusion signal per cell (arrows) was considered as *EWSR1-ATF1* fusion positive (Case 1, FISH×1000)

The FISH detection results are shown in Table 3. *EWSR1* rearrangement (Case 1; Fig. 6a) was positive in all cases. Four cases (Cases 1, 2, 3, and 4) were positive (4/5) for *EWSR1–ATF1* fusion (Case 1; Fig. 6b). *MAML2* rearrangement was tested in three cases (Cases 7, 9, and 10), all of which showed no re-arrangement (0/3, not shown). The FISH assay revealed *EWSR1* rearrangement in the CCC case with HGT, and this molecular alteration was confirmed in both conventional and HGT regions.

**Table 3:** FISH detection results in 10 cases of CCC

NO	Site	*EWSR1*	*EWSR1-ATF1*	*MAML2*
1	Right submandibular gland	+	+	ND
2	Nasal cavity	+	+	ND
3	Left tonsil	+	+	ND
4	Left maxillary sinus	+	+	ND
5	Root of the tongue	+	ND	ND
6	Base of tongue	+	–	ND
7	Right hard palate	+	ND	–
8	Right hard palate	+	ND	ND
9	Right hard palate	+	ND	–
10	Left hard palate	+	ND	–

*FISH* fluorescence in situ hybridization, *ND* not done

## Discussion

In the 2017 WHO classification of head and neck tumors, CCC was defined as a low-grade salivary gland malignancy composed of tumor cells with clear cytoplasm [2], rather than as a clear cell carcinoma. It has been classified as “not otherwise specified” (NOS), as it lacks clear clinicopathological features and was initially considered a diagnosis of exclusion in the 2005 WHO classification. Recent molecular genetic data has gradually revealed the genetic background of CCC and enabled greater understanding and broadening of its morphological spectrum, which has contributed to its removal from the NOS classification and increased its recognition as a rare malignancy that can undergo HGT and occur at uncommon sites.

The clinical symptoms of CCC depend on the lesion location, and the main clinical symptoms in our cohort included the development of a painless, firm mass, epistaxis, paresthesia, ulceration, and dysphagia. When CCC appears as a painless mass, it is typically ignored by patients. Case 1 documented a painless mass arising in the right submandibular gland, which was considered benign after fine needle aspiration, so no further surgery was performed. After an uneventful 20 years, the mass began to enlarge rapidly and painfully, and was diagnosed as CCC with HGT after resection. Therefore, the rapid enlargement of a longstanding tumor suggests the possibility of increased malignancy, especially if accompanied by a sudden onset of pain. To our knowledge, this is the first case of HGT detected by FISH testing, arising in the submandibular gland after such a long history.

HGT is a rare event that has been described in a variety of salivary gland malignancies, including adenoid cystic carcinoma, acinic cell carcinoma, epithelial–myoepithelial carcinoma (EMC), and low-grade MEC [11–14]. HGT is an exceedingly rare phenomenon in CCC; only two cases of HGT [6, 7] have been reported so far, and only one has been confirmed by molecular testing [7]. The clinicopathological and molecular features of the cases previously published and of the current HGT case are summarized as follows. First, HGT may occur at the initial presentation or result from malignant progression in patients with a longstanding disease course. Second, it exhibits aggressive clinical behavior, and the lung is a common metastatic site. Third, focal regions, at least, display moderate to severe nuclear aplasia, frequent or atypical mitotic figures, and necrosis. Fourth, conventional and HGT regions coexist, and both components share the same molecular alterations (*EWSR1* rearrangement), providing reliable evidence that the HGT component represents a transformation of CCC, rather than the coexistence with another SGT. However, it is noteworthy that not all CCCs with aggressive behavior show HGT or poorly differentiated morphologies, and there is currently no histological grading system to predict the aggressive behavior in tumors [15], making further investigation worthwhile. In HGT cases, p53 showed wild type expression in HGT areas, inconsistent with previous reports where adenoid cystic carcinoma and MEC with HGT showed mutant type expression patterns for this marker [11, 16], suggesting that the mechanism by which HGT occurs in CCC needs further investigation.

CCC was initially termed HCCC due to the prominent stromal hyalinization. Although the stroma of most CCCs exhibits hyalinization, CCC does not always present with this morphology, sometimes exhibiting a fibrocyte-rich stroma or loose mucinous matrix in some cases. For example, the neoplastic stroma was fibrous with focal mucinous changes in Cases 2, 3, and 9. CCC was adopted as the formal name by the 2017 WHO classification of head and neck tumors, with HCCC as its synonym. We suggest that it is appropriate to include HCCC as a subtype of CCC, as these tumors share the same molecular alterations (*EWSR1* rearrangement), regardless of whether their stroma are hyalinized.

Advances in the molecular genetics of SGTs have facilitated the discovery of other genes as the novel *EWSR1* fusion partners in CCCs. Although it is well documented that the *EWSR1–ATF1* fusion occurs in most CCCs [1, 2], a small proportion of cases showed other molecular rearrangements; notably, Chapman et al. [17] recently described three cases with *EWSR1–CREM* fusion. In our cohort, all cases had *EWSR1* rearrangements, as confirmed by FISH testing, and four of five cases harbored *EWSR1–ATF1* fusions, while one case did not, suggesting that *EWSR1* might have had another partner gene.

CCCs show a predilection for the oral cavity. However, it is important to note that this cancer can also occur in uncommon sites, such as the nasal cavity, left tonsil, and left maxillary sinus, as observed in our cohort (Cases 2, 3, and 4). Approximately 50 cases of CCC at these uncommon sites have been reported in the English literature, but some cases have not been genetically confirmed [18–22]. The differential diagnosis of CCCs arising in these rare sites is different from that of tumors occurring at common sites. Thus, a greater general awareness of rare tumors arising in uncommon sites is required, as these are particularly susceptible to misdiagnosis.

Differential diagnosis is very challenging when CCCs occur at uncommon sites. In Case 2, CCC arising in the nasal cavity had to be differentiated from renal cell-like sinonasal adenocarcinoma, which is composed of glands lined by cuboidal cells with clear cytoplasm. However, Case 2 was positive for p63, p40, and CK5/6 and harbored the *EWSR1* translocation, characteristics which renal cell-like sinonasal carcinoma does not express. Case 3 involved the left tonsil and needed to be differentiated from human papilloma virus (HPV)-related squamous cell carcinoma (SCC), which occurs more frequently in the oropharynx.

The use of p16 IHC staining, typically used as a surrogate marker for HPV in SCC of the head and neck, to distinguish between tumor types would likely lead to misdiagnosis. Bishop et al. [23] reported that p16 is frequently expressed in CCC, with more than 70% of tumor cells staining positive in some tumors. Therefore, p16 staining positivity should not be considered a useful tool to distinguish between CCC and HPV-related SCC. Fortunately, p16 staining was negative in our case, so we did not need further HPV RNA testing. Case 3, arising in the maxillary sinus, needed to be differentiated from clear cell variant squamous cell carcinoma (CCSCC) because it demonstrated a squamous immunophenotype; however, it lacked squamous dysplasia/carcinoma-in-situ and exhibited less nuclear pleomorphism than in CCSCC. Thus, testing for *EWSR1* rearrangement and/or *EWSR1–ATF1* fusion could be helpful in cases at uncommon sites.

In addition to the above differential diagnoses, CCCs can be differentiated from other clear cell rich-SGTs. In the case of clear cell MC, discrimination is essential because MCs with *EWSR1* rearrangement exhibit aggressive clinical behaviors and have poor clinical outcomes compared with CCCs [24]. Traditionally, *EWSR1* rearrangements were considered specific to CCCs but have since been confirmed in MCs [24, 25]. Moreover, CCCs were initially believed to be negative for myoepithelial markers; however, a minor subset of CCCs confirmed by *ATF1* rearrangement exhibited S-100 and/or SOX10 immunoreactivity [25]. Thus, distinguishing between CCCs expressing myoepithelial markers and MCs with *ATF1* rearrangements requires further study. Fortunately, all cases here were negative for a panel of myoepithelial markers (SMA, calponin, S-100 protein, GFAP, and CD10). In addition, SOX10, a transcription factor that plays a key role in the differentiation of the neural crest, maintenance of Schwann cells and melanocytes, and that was recently found to be expressed in myoepithelial tumors [26], was also not detected. These results virtually excluded the possibility of MC.

An additional SGT that warrants differential diagnosis is MEC. Mucicarmine negativity is typically used to distinguish CCC from clear cell variant MEC. However, intracellular mucin may be present in approximately 30% of CCC tumors [2, 27]. Fortunately, these two tumor types have different molecular alterations, with MECs typically exhibiting MAML2–CRTC1 or MAML2–CRTC3 fusion. Cases 7, 9, and 10 were submitted for confirmation by molecular testing, with a preliminary diagnosis of MEC. However, *MAML2* was intact, and *EWSR1* translocation was detected, confirming the diagnosis for CCC. These three cases correspond with previous reports that MECs with *EWSR1* rearrangements but no *MAML2* fusion were in fact CCCs [27]. Moreover, tumors in cases 7 and 10 arose from the overlying mucosal epithelium, which has never been described for MEC, and may aid in distinguishing these tumor types. EMC, another SGT, resembles CCC when it is predominantly composed of clear cells; however, CCC lacks the biphasic pattern of EMC. In addition, *HRAS* mutations have a high diagnostic value for EMC [28] but not CCC.

Importantly, CCCs should be differentiated from odontogenic tumors. *EWSR1–ATF1* and *EWSR1–CREM* fusions are found in both CCC and clear cell odontogenic carcinoma (CCOC) [22, 29–32], and there is overlap in histomorphology and immunophenotypes, with the exception that CCOC occurs in the jaw. These findings highlight the biological link between them, and whether they are the same entity occurring at different locations is still under debate. In our cohort, the possibility of CCOC was excluded by imaging analysis.

In addition to primary tumors, metastatic clear cell renal cell carcinoma needs to be ruled out. The renal tumor is immunoreactive for RCC (renal cell carcinoma marker), PAX8, and CD10, which were all negative in the CCCs investigated here.

CCC mostly behaves indolently, and treatment typically involves complete excision. Postoperative radiotherapy can be used in patients with positive margins, recurrent or more aggressive disease, or HGT [6, 7, 18]. However, the efficacy of radiotherapy and/or chemotherapy is unclear, although some patients have received these treatments [33]. Case 1 (HGT) received postoperative chemotherapy. Cases 4, 8, and 10 received radiotherapy after surgery, without evidence of disease at follow-ups of 88, 11, and 54 months, respectively. The remaining five patients remained disease-free at 8- and 80-month follow-ups, despite having received no additional therapy after surgery. Therefore, the prognosis of CCC is generally good, but the late onset of local recurrence and distant metastases have been sporadically recorded [34–36], suggesting the need for long-term follow-ups.

The present study has certain limitations. Comprehensive molecular studies should be performed, and alternative next-generation sequencing techniques may, for example, detect other partner genes of *EWSR1* in addition to *ATF1* and *CREM*. However, it is generally believed that a definitive diagnosis can be made by combining salient histological features, immunophenotypes, and molecular testing.

In summary, the general awareness of CCCs with HGT and those arising in uncommon locations needs to be increased, as these are particularly likely to be misdiagnosed. Sudden and rapid expansion of a longstanding CCC case should be a forewarning of the possibility of HGT. Our results showing the same immunophenotypic and molecular alterations (*EWSR1* rearrangement) in the HGT area and conventional areas of CCC provide reliable evidence that the HGT component represented a transformation of CCC rather than the coexistence of another SGT. CCCs need to be differentiated from many other clear cell-rich tumors, and their diagnosis needs to be confirmed by combining morphology-based IHC staining with molecular detection. Notably, CCCs do not always have a hyalinized stroma, which has led to the proposal of using the term HCCC to denote a subtype of CCC. Finally, CCCs generally have a good prognosis according to our follow-up data, except for the HGT case, which needs continued treatment.

## Data Availability

The datasets used are available from the corresponding author on reasonable request.

## Abbreviations

CCC: Clear cell carcinoma
HGT: High-grade transformation
IHC: Immunohistochemistry
FISH: Fluorescence in situ hybridization
HCCC: Hyalinizing clear cell carcinoma
SGT: Salivary gland tumor
MEC: Mucoepidermoid carcinoma
WHO: World Health Organization
HE: Hematoxylin and eosin
FFPE: Formalin-fixed paraffin-embedded
CT: Computed tomography
SCC: Squamous cell carcinoma
CCSCC: Clear cell variant squamous cell carcinoma
MC: Myoepithelial carcinoma
CCOC: Clear cell odontogenic carcinoma
